# Cost comparison of microscopy vs. empiric treatment for malaria in southwestern nigeria: a prospective study

**DOI:** 10.1186/1475-2875-9-371

**Published:** 2010-12-22

**Authors:** Ravi Parikh, Isaac Amole, Margaret Tarpley, Daniel Gbadero, Mario Davidson, Sten H Vermund

**Affiliations:** 1 Institute for Global Health, Vanderbilt University School of Medicine, 2215 Garland Ave. (LH319), Nashville, TN 37232-0242, USA; 2 Baptist Medical Centre/Bowen University Teaching Hospital, Ogbomoso, Oyo State, Nigeria; 3 Department of Surgery, Vanderbilt University School of Medicine, Nashville, TN, USA; 4 Department of Biostatistics, Vanderbilt University School of Medicine, Nashville, TN, USA; 5 Department of Pediatrics, Vanderbilt University School of Medicine, Nashville, TN, USA

## Abstract

**Background:**

Presumptive treatment for malaria is common in resource-limited settings, yet controversial given the imprecision of clinical diagnosis. The researchers compared costs of diagnosis and drugs for two strategies: (1) empirical treatment of malaria via clinical diagnosis; and (2) empirical diagnosis followed by treatment only with Giemsa smear confirmation.

**Methods:**

Patients with a diagnosis of clinical malaria were recruited from a mission/university teaching hospital in southwestern Nigeria. The patients underwent free Giemsa thick (diagnosis) and thin (differentiation) smears, but paid for all anti-malarial drugs. Clinical diagnosis was made on clinicians' judgments based on symptoms, including fever, diarrhoea, headache, and body aches. The paediatric regimen was artesunate (6-9 tablets of 3 mg/kg on day one and 1.5 mg/kg for the next four days) plus amodiaquine (10 mg/kg day 1-2 and 5 mg/kg on day three in suspension). Adults were given two treatment options: option one (four and one-half 50 mg artesunate tablets on day one and nine tablets for the next four days, plus three 500 mg sulphadoxine/25 mg pyrimethamine tablets) and option two (same artesunate regimen plus nine 200 mg tablets of amodiaquine at 10 mg/kg day 1-2 and 5 mg/kg on day three). The researchers calculated the costs of smears/drugs from standard hospital charges.

**Results:**

Doctors diagnosed 304 patients (170 adults ages >16 years and 134 pediatric) with clinical malaria, prescribing antimalarial drugs to all. Giemsa thick smears were positive in 115/304 (38%). The typical patient cost for a Giemsa smear was 550 Naira (US$3.74 in 2009). For children, the cost of testing all, but treating only Giemsa positives was N888 ($6.04)/child; the cost of empiric treatment of all who were clinically diagnosed was lower, N660 ($4.49)/child. For adults, the cost of testing all, but treating only Giemsa positives was N711 ($4.84)/adult for treatment option one (artesunate and sulphadoxine/pyrimethamine) and N730 ($4.97)/adult for option two (artesunate and amodiaquine). This contrasts to lower costs of empiric treatment for both options one (N610 = $4.14/adult) and two (N680=$4.63/adult).

**Conclusions:**

Empiric treatment of all suspected cases of malaria was cheaper (at the end of the dry to the beginning of the rainy season) than only treating those who had microscopy-confirmed diagnoses of malaria, even though the majority of patients suspected to have malaria were negative via microscopy. One can acknowledge that giving many malaria-uninfected Nigerians anti-malarial drugs is undesirable for both their personal health and fears of drug resistance with overuse. Therefore, funding of rapid diagnostic tests whose performance exceeds the Giemsa smear is needed to achieve an ideal of diagnostic confirmation before treatment.

## Background

Empiric treatment for malaria is the norm in many resource-limited settings, and is usually based upon non-specific findings such as fever, body aches, diarrhoea, and splenomegaly (especially in children). Empiric treatment remains controversial since many patients who do not have malaria are treated as such [1,2]. Since Nigerian health care is largely fee-for-service, the additive cost of laboratory tests along with the cost of drugs may be prohibitive for malaria patients. Nigerian patients typically ask for treatment and forego a blood test. Malaria is Nigeria's leading cause of morbidity, and the direct loss to the economy of malaria infections is ≈132 billion Naira (US$898 billion at 147 Naira per U.S. dollar) [3,4]. The current Nigerian "gold standard" is the Giemsa thick smear; newer rapid diagnostic tests (RDTs) give convenient and quick results, but are expensive and sometimes less sensitive [5-8].

The researchers determined how many of the empirically treated malaria cases were positive by Giemsa stain and microscopy. The researchers then assessed whether it would be more cost-effective to treat everyone empirically versus testing everyone with suspected malaria and treating those who are positive on Giemsa smear.

## Methods

All patients who were clinically diagnosed with malaria and treated at Baptist Medical Centre (also known as the Bowen University Teaching Hospital) in Ogbomoso, Nigeria (northern Oyo state) were enrolled in this study from May 23 to July 13, 2009. This corresponds to the tail end of dry season and the beginning of the rainy season. The researchers' patients presented at three of four primary care sites in the hospital: the adult outpatient clinic, the paediatric outpatient clinic, and the emergency room. The study did not enroll in the obstetrics/gynaecology clinic.

The researchers provided free malaria testing using Giemsa stains to all patients (adult and paediatric), comparing results with empirical diagnoses based on a doctor's history and physical examination (nurses and clinical officers did not make final diagnoses). Clinicians suspected malaria when patients had a constellation of fever, body- and headaches, splenomegaly (children), and/or diarrhoea. No training for malaria diagnosis was given.

A certified haematologist who had received recent WHO malaria diagnosis training reviewed the smears. Due to lax pharmaceutical law enforcement, many drugs (or counterfeits) can be obtained over-the-counter. The researchers recorded whether or not patients took any prior medical treatment for malaria in order to judge the possibility of false negative results. The cost for each smear was based upon charges to patients at the hospital, and the supplies for these smears are listed (Table 1).

**Table 1:** Materials and costs for laboratory diagnoses by Giemsa staining at the Baptist Medical Centre/Bowen University, 2009.

Cost of Supplies in bulk	Cost of materials for each test	Materials used for a Giemsa thick smear	Materials used for a Giemsa thin smear
▪ Methanol: N5,500/2.5L ▪ Slides: N200/50 slides ▪ EDTA tube: N21,000/1500 ▪ (Blood lancets) N600/200 ▪ Syringes and Needles N1800/100 ▪ Giemsa Stain N3500/500 mL ▪ Buffer salts (Disodium hydrogen orthophosphate and potassium dihydrogen orthophosphate) N3500/kg ▪ Cover Slips: N250/100 ▪ Immersion Oil: N2,500/100 mL	▪ EDTA tube N14 ▪ Giemsa Stain: N3.11 ▪ Methanol fix N8.8 ▪ Slide: N4 ▪ Cover slip: N2.5 ▪ Syringe: N18 ▪ Buffer (Giemsa): N0.26 ▪ Lancet: N3	▪ 4 mL buffer (1 part stain to 9 parts buffer) ▪ 0.44 mL Giemsa Stain ▪ 4 mL of Buffer to wash slide ▪ 0.05 mL of oil for microscope viewing ▪ 1 EDTA Tube ▪ 1 Slide ▪ 1 Cover Slip ▪ 1 syringe and needle	▪ 4 mL buffer (1 part stain to 9 parts buffer) ▪ 0.44 mL Giemsa Stain ▪ 4 mL of Buffer to wash slide ▪ 0.05 mL of oil for microscope viewing ▪ 4 mL Methanol for fixation ▪ 1 EDTA Tube ▪ 1 Slide ▪ 1 Cover Slip ▪ 1 syringe and needle

Note: N. stands for Naira, the Nigerian currency. EDTA stands for ethylenediaminetetraacetic acid, a blood preservative.

Patients had to pay for their own medicines, as would normally be the case. The paediatric regimen was artesunate (6-9 tablets of 3 mg/kg on day one and 1.5 mg/kg for the next four days) plus amodiaquine (10 mg/kg on days one to two and 5 mg/kg on day three in suspension). Adults were given two treatment options: option one (four and one-half 50 mg artesunate tablets on day one and nine additional artesunate tablets over the next four days, plus three 500 mg sulphadoxine/25 mg pyrimethamine tablets) and option two (four and one-half 50 mg artesunate tablets on day one and nine additional artesunate tablets for the next four days plus nine 200 mg tablets of amodiaquine at a dose of 10 mg/kg on day one to two and 5 mg/kg on day three). As with diagnostics, the researchers calculated the costs of drugs from these standard medical center charges. Chi square tests were used for testing statistical significance.

## Results and Discussion

Clinicians diagnosed 304 patients (170 adults and 134 children under 16) with presumptive malaria; all were treated. Of the 304 tests, 115 were Giemsa thick smear positive (38%) and 189 (62%) were negative (none with missing data Table 2). Of the 304 patients, 99 (33%) had taken anti-malarials after the onset of symptoms, but prior to presentation in the clinic.

**Table 2:** Comparison of Adult and Pediatric Patients

	Adult (N = 170)	Pediatric (N = 134)	P-value
Consumed antimalarials prior to visit	28% (47)	39% (52)	0.039
Did not consume prior antimalarials	72% (123)	61% (82)	
Female	61% (104)	40% (54)	<0.001
Male	39% (66)	60% (80)	
Giemsa Stain Blood Smear Negative (-)	74% (125)	48% (64)	<0.001
Giemsa Stain Blood Smear Positive (+)	26% (45)	52% (70)	

Note: P values using Chi-square test.

Of the 170 adults, 47 (28%) presented having taken some type of anti-malarial drug. Among persons who had taken anti-malarials prior to clinical presentation, 26% (12/47) were Giemsa smear positive, compared to 27% (33/123) of those who had not taken anti-malarials (p = 0.9, Table 3). Of the 134 children, 52 (39%) were reported to have taken anti-malarial drugs prior to presentation, while 82 (61%) had not. Among the children who consumed anti-malarials prior to presentation, 38% (20/52) were Giemsa smear positive, compared to 61% (50/82) of children who had not taken anti-malarials (p = 0.01, Table 4).

**Table 3:** Summary of Giemsa Results for Adults based upon Consumption of Previous Antimalarials

	Consumed Antimalarials Prior to Presentation N = 47	Did Not Consume Antimalarials Prior to Presentation N = 123
Giemsa Stain Blood Smear Negative (-)	74% (35)	73% (90)
Giemsa Stain Blood Smear Positive (+)	26% (12)	27% (33)

Note: P = 0.9 using Chi-square test.

**Table 4:** Summary of Giemsa Results for Pediatric Patients based upon Consumption of Previous Antimalarials

	Consumed Antimalarials Prior to Presentation N = 52	Did Not Consume Antimalarials Prior to Presentation N = 82
Giemsa Stain Blood Smear Negative (-)	62% (32)	39% (32)
Giemsa Stain Blood Smear Positive (+)	38% (20)	61% (50)

Note: P = 0.01 using Chi-square test.

The patient cost of one Giemsa smear was 550 Naira (US$3.74 in 2009). The cost of testing all and treating only Giemsa positive children was N888 (US$6.04)/paediatric patient; empiric treatment of clinically diagnosed children was N660 (US$4.49)/paediatric patient. The cost of testing all and treating only Giemsa positive adults was N711 (US$4.84)/adult for treatment option one and N730 (US$4.97)/adult for option two. The respective costs for empiric treatment of all adult patients for treatment option one was N610 (US$4.14)/adult and N680 (US$4.63)/adult for option two.

Giemsa thin smears were done on all patients, demonstrating that each positive Giemsa thick smear was a result of *Plasmodium falciparum*. By varying the theoretical costs of diagnostic tests, the researchers found that for the treatment costs to start to favour a Giemsa diagnosis for therapy, Giemsa charges at the hospital would have to drop from N550 (US$3.74) to N448 (US$3.05) and N500 (US$3.40) for adults using treatment options one and two, respectively. For children, the Giemsa charge would have to drop further to N318 (US$2.16). The study did not consider false negative Giemsa smears for very low parasitaemia levels in the model.

The researchers found that empirical treatment of those clinically diagnosed with malaria was more cost effective than testing everyone with Giemsa and then treating only those who were smear-positive. Due to limited resources in most malarious countries and the patient costs and logistical obstacles of the diagnostic testing, it is easy to understand that presumptive therapy remains the backbone of most malaria control efforts in Africa. Saving money is not the only consideration; more extensive antimalarial use may stimulate inappropriate use and drug resistance. Hence, the availability of inexpensive rapid drug tests to achieve high sensitivity and high specificity in diagnosis would be optimal if the diagnostic costs were roughly offset by the savings in unnecessary therapy. If hospital charges dropped by 18.4% for treatment option one and 9.1% for treatment option two, then treating only Giemsa positive adults would be cost-effective. For children, Giemsa smear charges would have to drop by 42.1% for children to favour the laboratory-based diagnostic strategy with current drug costs. The fact that this Nigerian mission hospital had more doctors than non-physician practitioners making malaria diagnoses is unlikely to affect the generalizability of these findings; costs used were those of the hospital, including provider salaries. If lower paid practitioners are doing the syndromic diagnosis of malaria elsewhere, the costs of empiric therapy are likely to be even more favorable than with laboratory confirmation.

A 2009 study in Nigeria reported the use of presumptive treatment to be more cost-effective than microscopy, noting that a "43.1% prevalence level showed an incremental cost effectiveness ration (ICER) of 221 per deaths averted between Rapid Diagnostic Tests (RDT) and presumptive treatment while microscopy is dominated at that level." The study did not examine the use of RDTs as they are not commonly used (or even available) in many Nigerian health care practices, and are more costly than Giemsa in any case. RDT-directed therapy may be a viable option when prevalence is lower and RDT costs are subsidized [9-13]. A 2008 study in Ghana found that under ideal field conditions and with low and moderate malaria prevalence levels, rapid diagnostic tests (RDTs) would be more cost beneficial than microscopy or empiric treatment. Interestingly, the authors suggested that under more "real world" operational conditions, "microscopy becomes more cost beneficial than rapid diagnostic tests [12]."

A strength of this study was that 100% of the medical records and needed data were secured for the 304 patients eligible for the study over the defined study period. This reflects an unusually good record system at the Baptist Medical Centre. A limitation of this study was that it was conducted at only one center serving an urban and peri-urban region of ≈1 million low income Nigerians, limiting the generalizabilty of the findings. This study does not include costs of anti-malarial treatments that persons might have taken before coming to the hospital.

## Conclusion

This study found that the least expensive approach to malaria treatment in Nigeria with direct real-world costs in 2009 was treatment of those suspected to have malaria based upon symptoms at the end of the dry to the beginning of the rainy season. However, most presumptive malaria treatments are going to Giemsa smear negative persons. One can acknowledge that giving many malaria-uninfected Nigerians anti-malarial drugs is undesirable for both personal health and fears of drug resistance with overuse. The ideal solution would be a simple, cheaper, sensitive, and specific malaria test that offers affordable rapid diagnosis to reduce false positive treatments without missing the diagnosis of malaria. In the meantime, it will be necessary for donors to fund both treatment and RDTs to achieve optimized drug utilization in Africa.

## Competing interests

The authors declare that they have no competing interests.

## Authors' contributions

RP formulated the design, carried out the project in Nigeria, and wrote the manuscript. IA oversaw the enrollment, clinical diagnosis, and treatment of malaria in adult patients. MT was involved with the IRB process, informed consent procedures, and provided logistical support to carry out the study. DG oversaw the enrollment, the clinical diagnosis and treatment of malaria in paediatric patients. MD performed the statistical analysis of the data. SV was involved in the design of the data analysis, and the writing and editing of the manuscript. All authors read and approved the final manuscript.
